# An infectious aetiology for childhood brain tumours? Evidence from space–time clustering and seasonality analyses

**DOI:** 10.1038/sj.bjc.6600228

**Published:** 2002-04-08

**Authors:** R J Q McNally, D P Cairns, O B Eden, F E Alexander, G M Taylor, A M Kelsey, J M Birch

**Affiliations:** Cancer Research UK Paediatric & Familial Cancer Research Group, Central Manchester and Manchester Children's University Hospitals NHS Trust, Manchester M27 4HA, UK; Academic Unit of Paediatric Oncology, Central Manchester and Manchester Children's University Hospitals NHS Trust, Hospital Road, Manchester M27 4HA, UK; Department of Public Health Sciences, The University of Edinburgh Medical School, Teviot Place, Edinburgh EH8 9AG, UK; Immunogenetics Laboratory, Department of Medical Genetics, St. Mary's Hospital, Manchester M13 0JH, UK; Department of Pathology, Central Manchester and Manchester Children's University Hospitals NHS Trust, Hospital Road, Manchester M27 4HA, UK

**Keywords:** brain tumours, children, aetiology, infection, seasonal variation, space–time clustering

## Abstract

To investigate whether infections or other environmental exposures may be involved in the aetiology of childhood central nervous system tumours, we have analysed for space–time clustering and seasonality using population-based data from the North West of England for the period 1954 to 1998. Knox tests for space–time interactions between cases were applied with fixed thresholds of close in space, <5 km, and close in time, <1 year apart. Addresses at birth and diagnosis were used. Tests were repeated replacing geographical distance with distance to the Nth nearest neighbour. N was chosen such that the mean distance was 5 km. Data were also examined by a second order procedure based on K-functions. Tests for heterogeneity and Edwards' test for sinusoidal variation were applied to examine changes of incidence with month of birth or diagnosis. There was strong evidence of space–time clustering, particularly involving cases of astrocytoma and ependymoma. Analyses of seasonal variation showed excesses of cases born in the late Autumn or Winter. Results are consistent with a role for infections in a proportion of cases from these diagnostic groups. Further studies are needed to identify putative infectious agents.

*British Journal of Cancer* (2002) **86**, 1070–1077. DOI: 10.1038/sj/bjc/6600228 www.bjcancer.com

© 2002 Cancer Research UK

## 

In the developed world central nervous system (CNS) tumours are the second most common group of malignancies in children (Parkin *et al*, 1998). The aetiology of childhood CNS tumours is far from clear. Heritable syndromes are the only established causes, but these account for a minority of cases (Bondy *et al*, 1991). A number of statistically significant associations with certain exposures have been noted from case–control studies, including: consumption of cured meats/fish during pregnancy; insecticides/pesticides; farm residence; and electro-magnetic fields (Little, 1999). However, there is inconsistency between studies, and relative risks were all small.

There has been much speculation about the role of certain viruses in human brain tumours (Barbanti-Brodano *et al*, 1997), but very few epidemiological studies have addressed the possibility of an infectious aetiology. If infections are involved in the aetiology of childhood brain tumours, the distribution of cases may be predicted to exhibit space–time clustering. Space–time clustering is said to occur when excess numbers of cases are observed within various small geographical locations, but only at limited points in time. The presence of seasonal variation would also provide evidence for an infectious aetiology. We have therefore examined incidence data from the Manchester Children's Tumour Registry (MCTR) for presence of space–time clustering and seasonal variation. This registry is population based with consistently high ascertainment and contains verified diagnostic data over a 45 year period (Birch, 1988). The aims of our study were to test predictions of space–time clustering and seasonal patterns which might arise as a result of infectious mechanisms and to distinguish between exposures around the times of birth and onset by using time/place of birth and diagnosis respectively.

## MATERIALS AND METHODS

All cases, diagnosed with a CNS tumour between the 1st January 1954 and 31st December 1998, and registered by the MCTR were analysed. Ordnance Survey (OS) eight-digit (i.e., four-digit Easting and four-digit Northing) grid references were allocated to each case with respect to addresses at time of birth and diagnosis, locating each address to within 0.1 km. The following diagnostic groups were specified *a priori* for analysis: (i) astrocytoma (comprising: pilocytic astrocytoma; and other astrocytoma); (ii) ependymoma; (iii) medulloblastoma and other PNET's; (iv) other CNS tumours; and (v) total CNS tumours (Kleihues and Cavenee, 2000).

The following aetiological hypotheses were tested: (1) A primary factor influencing geographical or temporal heterogeneity of incidence of childhood CNS tumours is related to exposure to an infectious or other similarly occurring environmental agent either relatively close to disease onset or in-utero/peri-natally. (2) Geographical or temporal heterogeneity of incidence of childhood CNS tumours is modulated by differences in susceptibility between males and females and patterns of exposure related to level of population density.

There are four possible space–time interactions between: (i) times and places of diagnosis; (ii) times and places of birth; (iii) time of diagnosis and place of birth; and (iv) time of birth and place of diagnosis. The interpretation of these interactions will depend on the extent of migration between birth and diagnosis among cases (Birch *et al*, 2000).

Knox (1964) space–time clustering tests were applied to the data with thresholds fixed, *a priori*, as: close in space, less than 5 km, and close in time, less than 1 year apart. These limits are somewhat arbitrary, but this problem is overcome by using the K-function method. For the Knox test, where the observed number of pairs which are both close in time and close in space [O], is greater than the expected number [E], this indicates a tendency for pairs of cases which are close in space to have similar times and *vice versa*. One sided tests were used to detect a significant interaction. The strength of interactions [S] was indicated by calculating [(O-E)/E]×100 counts of pairs which are close in space and close in time. ‘Strength’ is a measure of the excess of observed cases over the expected. There is no theoretical maximum or minimum. For example, a value of 100% implies that O=2E, whilst a negative value indicates a situation where O is less than E. To adjust for the effects of different population densities, the tests were repeated replacing geographical distance thresholds by distance to the Nth nearest neighbour (*n*=31 for birth locations and 30 for diagnosis locations), using all locations of all the cases in the data set except addresses for the same child at a different time. N was chosen such that the mean distance was 5 km.

Two problems are apparent with the Knox test. First, boundary problems may be important since it can be impossible, or less probable, for some cases to be close in one dimension to other cases. The second problem concerns the arbitrariness of the thresholds chosen, which often results in multiple testing. A simplification, avoiding adjustment for boundary conditions, of a second order procedure based on K-functions (Diggle *et al*, 1995) is used in the present analyses to overcome the problem of multiple testing. Nearest neighbour (NN) approaches were also used as described above in relation to classical Knox tests.

The primary analysis was restricted to the main diagnostic groups. Only those diagnostic groups which exhibited space–time clustering with a significance level of *P*<0.1 for the total cases in the specified diagnostic group, using either the geographical distance or the NN threshold variations of the K-function method, were considered further. Such groups were split down by sub-diagnostic group as appropriate and were also examined for cross-clustering between cases from different groups or sub-groups. Further, those groupings which exhibited statistically significant space–time clustering, at the *P*=0.05 level, for the total cases in the specified diagnostic group, using either the geographical distance or NN threshold variations of the K-function method were analysed within age and gender subgroups, by level of population density, and also for the presence of seasonal variation.

Two age-groups (0–4 and 5–14 years) were chosen in order to differentiate between possible pre-natal and post-natal aetiological exposures. That is, positive results for the 0–4-year-olds may indicate the involvement of pre-natal exposures, whereas positive results for the 5–14-year-olds are more likely to indicate the involvement of post-natal exposures. Analysis by gender proceeded by examining ‘male : any’ and ‘female : any’ clustering pairs.

Using internal methods, addresses were classified as being located in a more densely populated area, or as being located in a less densely populated area. Analysis by population density was undertaken by considering ‘more densely populated : any’ and ‘less densely populated : any’ clustering pairs. It should be noted that these analyses (especially the analyses of ‘less densely populated: any’ clustering pairs) are potentially subject to a strong diluting influence from edge effects since neither the ‘more densely populated’ areas nor the ‘less densely populated’ areas form a single spatially contiguous zone.

To examine seasonality the cases were examined for monthly variation in birth and diagnosis dates using: (i) a chi-squared test for heterogeneity, and (ii) Edwards' test (Edwards, 1961) for sinusoidal variation. All cases of malignancies recorded by the MCTR, within the defined area, from 1954 to 1998, were used to examine overall monthly variation in the distribution of birth dates and diagnosis dates. These overall case distributions were then used to correct the case distributions of birth dates and diagnosis dates, respectively, for the CNS tumours and sub-diagnostic groups.

## RESULTS

The study included 422 astrocytomas (comprising 259 cases of pilocytic astrocytoma; and 163 cases of other astrocytoma), 109 ependymomas, 200 medulloblastomas and other PNET's, and 314 other CNS tumours. All cases were histologically verified except 10 which were classified as pilocytic astrocytoma of optic nerve on the basis of characteristic radiological and clinical evidence and 134 radiologically diagnosed tumours mainly of the brain stem and third ventricle. The latter were classified with ‘other CNS tumours’. Table 1

**Table 1:**
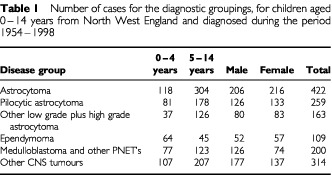
Number of cases for the diagnostic groupings, for children aged 0–14 years from North West England and diagnosed during the period 1954–1998

shows the numbers of males and females and children aged 0–4 and 5–14 years in each diagnostic group. Most of the evidence of space–time clustering occurred using place and time of diagnosis (Tables 2

**Table 2:**
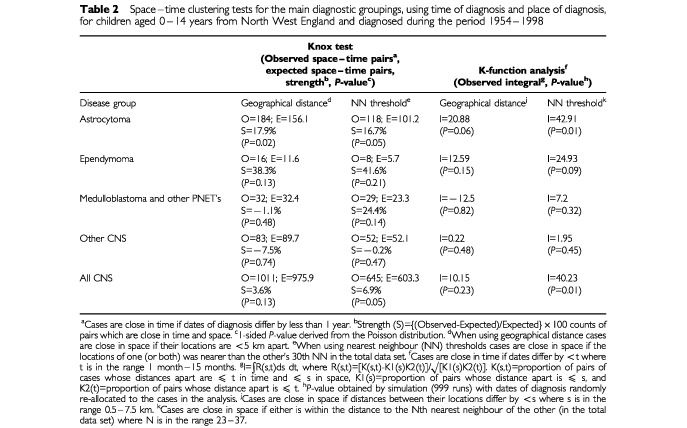
Space–time clustering tests for the main diagnostic groupings, using time of diagnosis and place of diagnosis, for children aged 0–14 years from North West England and diagnosed during the period 1954–1998

–6

**Table 6:**
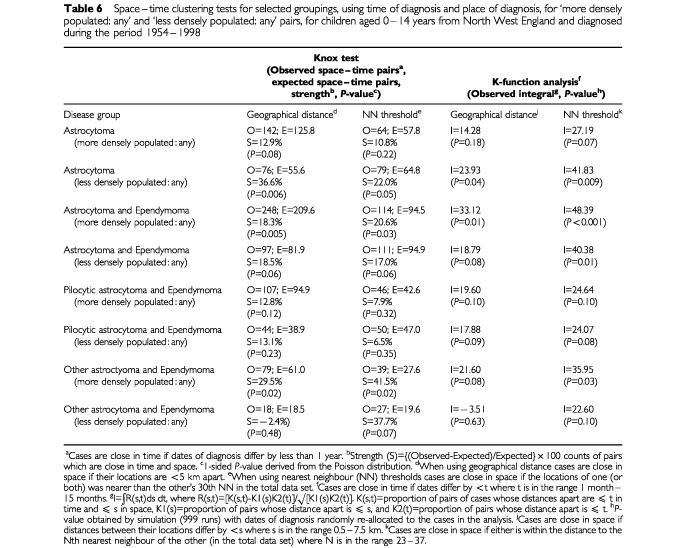
Space–time clustering tests for selected groupings, using time of diagnosis and place of diagnosis, for ‘more densely populated: any’ and ‘less densely populated: any’ pairs, for children aged 0–14 years from North West England and diagnosed during the period 1954–1998

), but there was some evidence of space–time clustering for certain diagnostic groups using place and time of birth as detailed below. There was no evidence for space–time clustering based on time of diagnosis and place of birth, and very weak evidence, for ependymoma only, based on time of birth and place of diagnosis (S=113.6%, *P*=0.05, using the NN threshold version of the Knox test, and I (observed value of the integral)=26.79, *P*=0.05, using the NN version of the K-function method).

Table 2 shows that three diagnostic groupings give *P*<0.1 using at least one of the four analysis methods and including a NN threshold version (astrocytoma; ependymoma; all CNS). There was no evidence of space–time clustering for other groups (*P*>0.1, using all four methods). In view of these results, it was decided to examine sub-groups of the astrocytomas (pilocytic astrocytoma; and other astrocytoma). Clustering implies involvement of environmental factor(s). Given the presence of clustering among cases of both astrocytoma and ependymoma, the possibility of common factor(s) is suggested. Therefore cross-clustering between astrocytomas and ependymomas was also examined.

Three diagnostic groupings show very striking evidence of space–time clustering (astrocytoma and ependymoma; pilocytic astrocytoma and ependymoma; other astrocytoma and ependymoma: *P*<0.05 using at least one of the four methods and including a NN threshold version) (Table 3

**Table 3:**
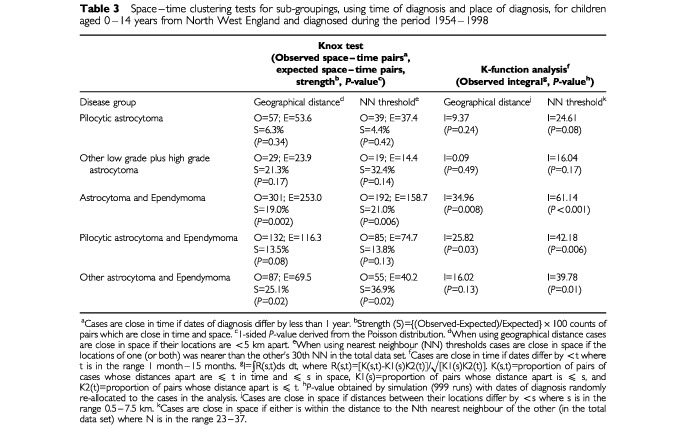
Space–time clustering tests for sub-groupings, using time of diagnosis and place of diagnosis, for children aged 0–14 years from North West England and diagnosed during the period 1954–1998

). These results show that cross-clustering of cases of astrocytoma with cases of ependymoma is frequent, in addition to clustering pairs of astrocytoma or ependymoma alone. In contrast, there was much less evidence for space–time clustering of cases of pilocytic astrocytoma and other astrocytoma by themselves.

The significant NN threshold analyses show that the clustering is not just a result of varying population density (Tables 2 and 3). Indeed, the K-function analyses show a consistent trend for stronger space–time clustering when adjustment for population density is made using the NN threshold approach.

The four diagnostic groups that showed most evidence for space–time clustering (astrocytoma; astrocytoma and ependymoma; pilocytic astrocytoma and ependymoma; other astrocytoma and ependymoma) were studied further.

First, they were divided into two age groups: 0–4 years and 5–14 years. Astrocytoma showed little evidence for clustering in either age group (Table 4

**Table 4:**
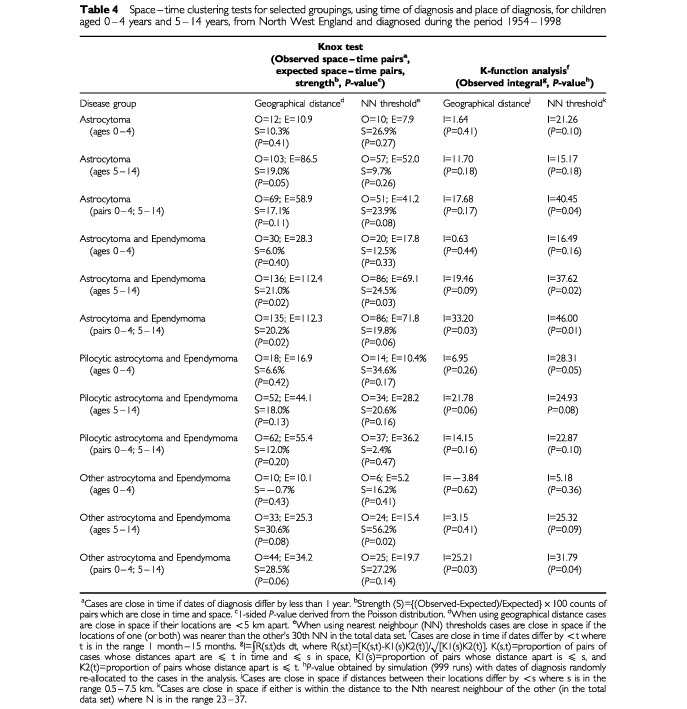
Space–time clustering tests for selected groupings, using time of diagnosis and place of diagnosis, for children aged 0–4 years and 5–14 years, from North West England and diagnosed during the period 1954–1998

). For the other three groups (astrocytoma and ependymoma; pilocytic astrocytoma and ependymoma; other astrocytoma and ependymoma) there was some evidence for clustering in cases aged 5–14 years (*P*<0.1 using at least one of the four methods and including a NN threshold version), but it was consistently less significant than for the entire age range, although the ‘strength of clustering’ was similar for some of the age groups. Further analyses showed marked cross-clustering between the younger and older cases for: astrocytoma; astrocytoma and ependymoma; and other astrocytoma and ependymoma, but not for pilocytic astrocytoma and ependymoma (see Table 4). Only one group showed evidence of space–time clustering in cases aged 0–4 years (pilocytic astrocytoma and ependymoma; *P*=0.05 using the NN threshold version of the K-function method). Therefore, in this specific group further analysis using time and place of birth was carried out. Results showed much stronger space–time clustering for this analysis (S=42.1%, *P*=0.0007, using the geographical distance version of the Knox test; S=28.5%, *P*=0.05, using the NN threshold version of the Knox test; I=27.2, *P*=0.03, using the geographical distance version of the K-function method, and I=45.01, *P*=0.01, using the NN threshold version of the K-function method).

For the four selected diagnostic groups, separate analyses were performed for ‘male : any’ and ‘female : any’ pairs (Table 5

**Table 5:**
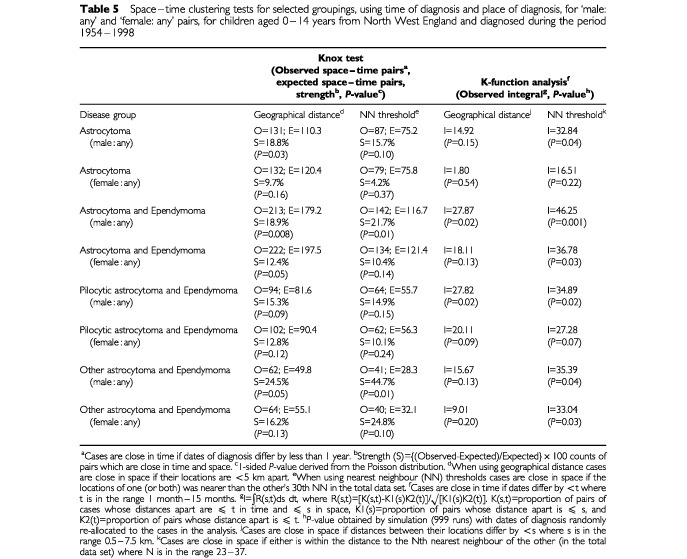
Space–time clustering tests for selected groupings, using time of diagnosis and place of diagnosis, for ‘male: any’ and ‘female: any’ pairs, for children aged 0–14 years from North West England and diagnosed during the period 1954–1998

). All groups exhibited significant evidence of space–time clustering (*P*<0.05) for ‘male : any’ pairs, using at least one of the four methods and including a NN threshold version. Only astrocytoma and ependymoma; and other astrocytoma and ependymoma showed significant space–time clustering for ‘female : any’ pairs, based on at least one of the four methods and including a NN threshold version. The strength of the clustering for ‘female : any’ pairs was consistently weaker. Further analysis of the pilocytic astrocytoma and ependymoma group, using time and place of birth, was carried out. Results showed much stronger space–time clustering for ‘male : any’ pairs than for ‘female : any’ pairs ((‘male : any’ pairs: S=58.2%, *P*=0.0001, using the geographical distance version of the Knox test; S=50.3%, *P*=0.007, using the NN threshold version of the Knox test; I=40.2, *P*=0.005, using the geographical distance version of the K-function method, and I=60.1, *P*<0.001, using the NN threshold version of the K-function method) and (‘female : any’ pairs: S=37.7%, *P*=0.007, using the geographical distance version of the Knox test; S=28.8%, *P*=0.08, using the NN threshold version of the Knox test; I=15.4, *P*=0.16, using the geographical distance version of the K-function method, and I=38.5, *P*=0.03, using the NN threshold version of the K-function method)).

Finally, separate analyses by population density were done (Table 6). Space–time clustering was stronger for ‘more densely populated : any’ pairs, except for cases of astrocytoma by themselves. Further analysis of the pilocytic astrocytoma and ependymoma group, using time and place of birth was carried out. Results showed statistically significant space–time clustering for ‘more densely populated : any’ pairs, but not for ‘less densely populated : any’ pairs ((‘more densely populated : any’ pairs: S=42.4%, *P*=0.002, using the geographical distance version of the Knox test; S=30.5%, *P*=0.10, using the NN threshold version of the Knox test; I=23.8, *P*=0.03, using the geographical distance version of the K-function method; and I=48.5, *P*=0.004, using the NN threshold version of the K-function method) and (‘less densely populated : any’ pairs : S=53.5%, *P*=0.02, using the geographical distance version of the Knox test; S=34.8%, *P*=0.07, using the NN threshold version of the Knox test; I=16.1, *P*=0.15, using the geographical distance version of the K-function method; and I=22.0, *P*=0.17, using the NN threshold version of the K-function method)).

For the analysis of seasonality based on month of diagnosis, the heterogeneity test detected a borderline significant departure from the uniform distribution in astrocytoma (*P*=0.09) and a highly significant departure for other CNS (*P*=0.001) (Table 7

**Table 7:**
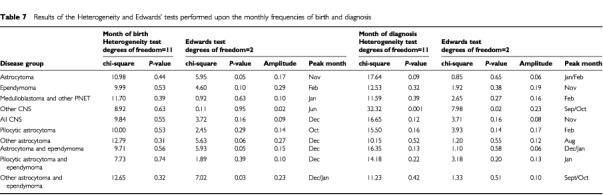
Results of the Heterogeneity and Edwards' tests performed upon the monthly frequencies of birth and diagnosis

). The Edwards' test only showed evidence for sinusoidal variation, based on month of diagnosis for other CNS (*P*=0.02). The results for this heterogeneous group may well be a chance occurrence. However, there was cyclical variation in month of diagnosis for craniopharyngioma (Edwards' test: *P*=0.04), but these findings are based on small numbers and should be treated with caution. Based on month of birth, the Edwards' test showed significant sinusoidal variation for three of the four selected groups (astrocytoma, *P*=0.05; astrocytoma and ependymoma, *P*=0.05; other astrocytoma and ependymoma, *P*=0.03) and borderline significance for ependymoma (*P*=0.10) and other astrocytoma (*P*=0.06). For these three groups, seasonal peaks in month of birth occurred in November, December and January, respectively.

## DISCUSSION

The analyses presented here have been performed using rigorous statistical methods on high quality incidence data. Space–time clustering and cross-clustering have been identified for specific diagnostic groups of CNS tumours involving astrocytoma and ependymoma. Additionally, seasonal patterns of incidence were also found amongst cases of astrocytoma and ependymoma, but only using month of birth.

The marked cross-clustering between cases of astrocytoma and ependymoma suggests shared aetiological factors. Although the cell of origin is different, paediatric astrocytoma and ependymoma do share certain characteristics. In particular, pilocytic astrocytoma and most ependymomas are both ‘slow growing’, low grade tumours and the majority of other astrocytomas in children are also of low grade. The similarities in growth pattern suggest the possibility of common mechanisms in the induction of these tumours.

Taken together, the space–time clustering and seasonal patterns provide consistent evidence of an environmental agent (or agents) in the aetiology of certain childhood astrocytomas and ependymomas. More specifically, there is support for two putative mechanisms:

1. a pre-natal or perinatal exposure to an environmental agent, contributing to the onset of pilocytic astrocytoma or ependymoma, after a variable latent period; and
2. a post-natal exposure to an environmental agent some time before diagnosis, contributing to the onset of astrocytoma or ependymoma, after a relatively short and constant latent period.

Environmental agents which may cause localised variations in incidence include airborne infections, electrical power lines, and factory emissions. However, the patterns of incidence, as exhibited by the finding of space–time clustering, with respect to time and place of diagnosis, are not consistent with a sustained exposure either geographically or over time. Thus, causative agents such as power lines and factory emissions are not supported by this analysis.

The agent responsible for the observed clustering is much more likely to exhibit a pattern of temporary occurrence at many points in time and space. Thus, infections are the most plausible aetiological agent that would explain space–time clustering patterns based on place and time of diagnosis found in the MCTR data.

The evidence for space–time clustering based on place and time of birth was weaker. One problem with the analyses by date of birth is that all cases born after 1938, but diagnosed before 1954 will be missed. Some cases may also be missed because they have not yet been diagnosed by the end of the study period (1998). However, the study period (1954–1998) is sufficiently long for such truncation to have little influence on the overall results. Indeed, at worst, the results based on date of birth, may be conservative.

The finding of stronger space–time clustering amongst ‘male : any’ pairs may be consistent with an infectious aetiology since males are more susceptible to infections (Washburn *et al*, 1965; Purtilo and Sullivan, 1979). The association of space–time clustering with more densely populated areas is also consistent with a role for infections. Closer proximity of individuals may allow infection to proliferate to a greater extent in densely populated areas than in less densely populated areas.

Two other epidemiological studies provide tentative direct evidence of the involvement of infections in childhood brain tumours. Linet *et al* (1996) reported an odds ratio of 2.4 in brain tumour cases compared with controls for infection in the neonatal period. Fear *et al* (2001) in a smaller study, but using similar methodology, found increased risk for childhood brain tumours associated with viral infections in pregnancy. In both studies the findings were not confined to specific infections but are consistent with the first mechanism proposed above. However, a case–control study of childhood malignancies found no excess risk associated with infections for CNS tumours, either during pregnancy or following birth (McKinney *et al*, 1999).

The only other study to apply formal statistical methods to population based incidence data on CNS tumours, from Sweden, did not find space–time clustering amongst cases of childhood astrocytoma (Hjalmars *et al*, 1999). There are a number of possible explanations for the apparent differences between the two studies. The lack of space–time clustering in Sweden may reflect: a different pattern or mechanism in terms of exposure to the relevant environmental agent(s); exposure to different agent(s); methodological differences; or the lower population density in Sweden.

In summary, we have found strong evidence of space–time clustering among certain diagnostic groupings of childhood CNS tumours, involving cases of astrocytoma and ependymoma. Seasonal variation in month of birth was also present. The results are consistent with a role for infections in at least a proportion of these cases, but with two distinct mechanisms, the first acting pre-natally (or around the time of birth), and the second acting post-natally (around the time of diagnosis). The evidence for the second mechanism is stronger than that for the first. The gender differences might suggest that males may be more susceptible than females. The possible involvement of infections is also given some support by the observed seasonality in birth dates.

While the suggestion of an infectious aetiology for childhood leukaemia is gaining momentum, scant attention has been paid to the possible involvement of infections in the aetiology of other childhood malignancies. Future studies should consider specific candidate infections and genetic markers of susceptibility.
